# Supplementary material for: Pseudomonas sp. COW3 Produces New Bananamide-Type Cyclic Lipopeptides with Antimicrobial Activity against Pythium myriotylum and Pyricularia oryzae
Source: Molecules. 2019 Nov 17;24(22):4170. doi: 10.3390/molecules24224170 (PMC6891508; doi:10.3390/molecules24224170)
Supplement: Supplementary file 1 [file molecules-24-04170-s001.pdf]

Supplementary materials

Pseudomonas sp. COW3 produces novel bananamide-type cyclic lipopeptides with antimicrobial activity against *Pythium myriotylum* and *Pyricularia oryzae*

Olumide Owolabi Omoboye[1,4], Niels Geudens[2], Matthieu Duban[3], Mickaël Chevalier[3], Christophe Flahaut[3], José C. Martins[2], Valérie Leclère[3], Feyisara Eyiwumi Oni[1&], Monica Höfte[1&*]

[1]Laboratory of Phytopathology, Department of Plants and Crops, Faculty of Bioscience Engineering, Ghent University, Coupure Links 653, B-9000 Ghent, Belgium; olumideowolabi.omoboye@ugent.be (OOO); FeyisaraEyiwumi.Olorunleke@UGent.be (F.E.O.); monica.hofte@ugent.be (M.H.)

[2]NMR and Structure Analysis Unit, Department of Organic and Macromolecular Chemistry, Faculty of Science, Ghent University, Krijgslaan 281, B-9000 Gent, Belgium; niels.geudens@ugent.be (N.G.); jose.martins@ugent.be (JM)

[3]Univ. Lille, INRA, ISA, Univ. Artois, Univ. Littoral Côte d'Opale, EA 7394-ICV- Institut Charles Viollette, F-59000 Lille, France; mickael.chevalier1@polytech-lille.fr (MC); matthieu.duban@univ-lille.fr (MD); christophe.flahaut@univ-artois.fr (CF); valerie.leclere@univ-lille.fr (VL)

[4]Department of Microbiology, Obafemi Awolowo University, 220005 Ile-Ife, Osun State, Nigeria; oomoboye@oauife.edu.ng (OOO)

*Corresponding author: Monica Höfte, E-mail address: monica.hofte@ugent.be; Phone: +32 9 264 60 17

[&]These authors share senior authorship

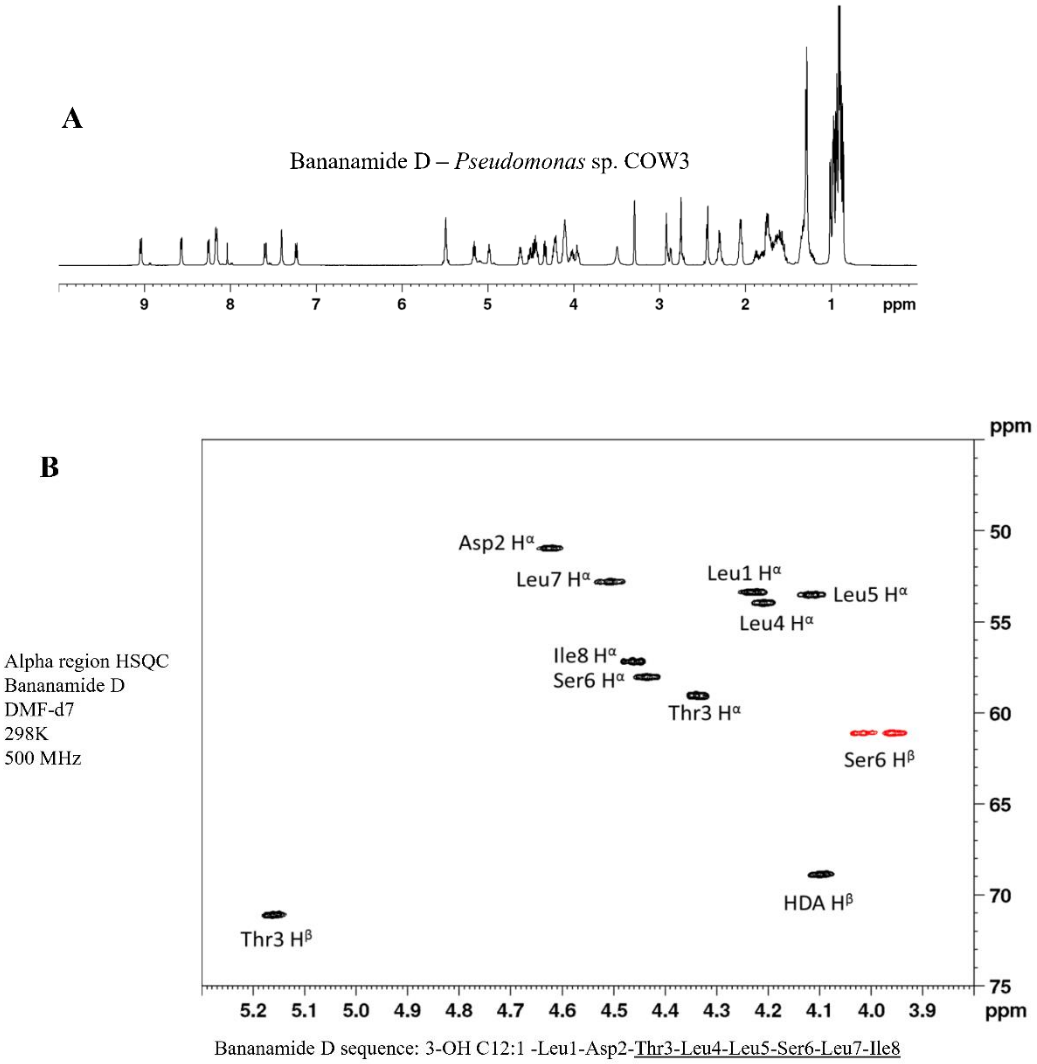

Figure S1. 1D ^{1}H NMR (**A**) and ^{1}H-^{13}C gHSQC spectrum (**B**) of bananamide D extracted from *Pseudomonas* sp. COW3 (DMF-d7, 298K, 500MHz). A. 1D ^{1}H NMR spectrum of the first main compound called bananamide D, eluting at 12.4 minutes. The presence of an unsaturation in the structure is immediately clear from the characteristic signal at 5.5 ppm. B. The alpha region of a ^{1}H-^{13}C gHSQC spectrum of the bananamide D shows the presence of 8 amino acids. The high chemical shift of the Thr CH^{β} indicates that the C-terminal ester bond is formed with this residue.

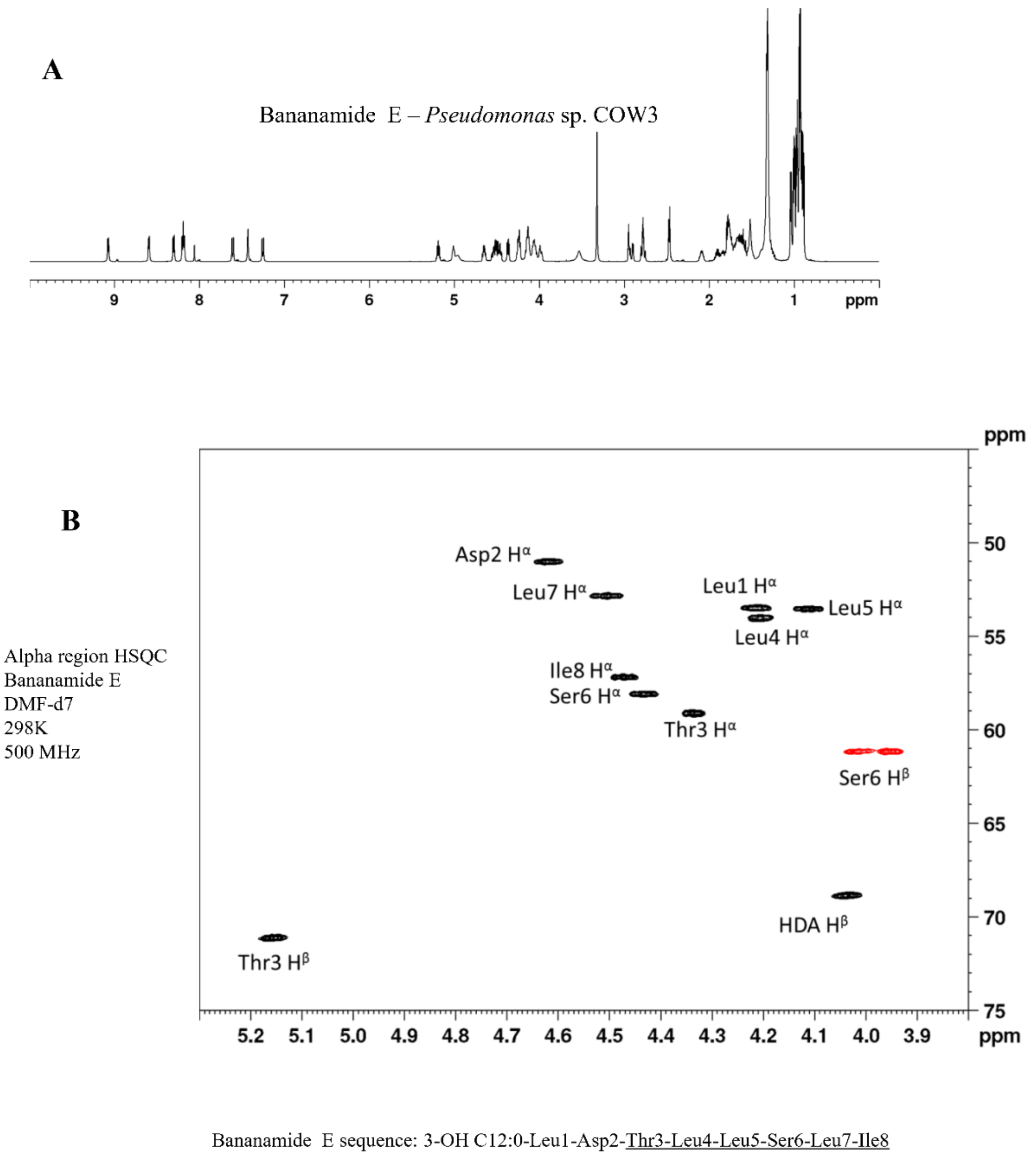

Figure S2. 1D ^{1}H NMR (**A**) and ^{1}H-^{13}C gHSQC spectrum (**B**) of bananamide E extracted from *Pseudomonas* sp. COW3 (DMF-d7, 298K, 500MHz). A. 1D ^{1}H NMR spectrum of the second main compound called bananamide E, eluting at 13.9 minutes. B. The alpha region of a ^{1}H-^{13}C gHSQC spectrum of bananamide E shows the presence of 8 amino acids. The high chemical shift of the Thr CH$^{\beta}$ indicates that the C-terminal ester bond is formed with this residue.

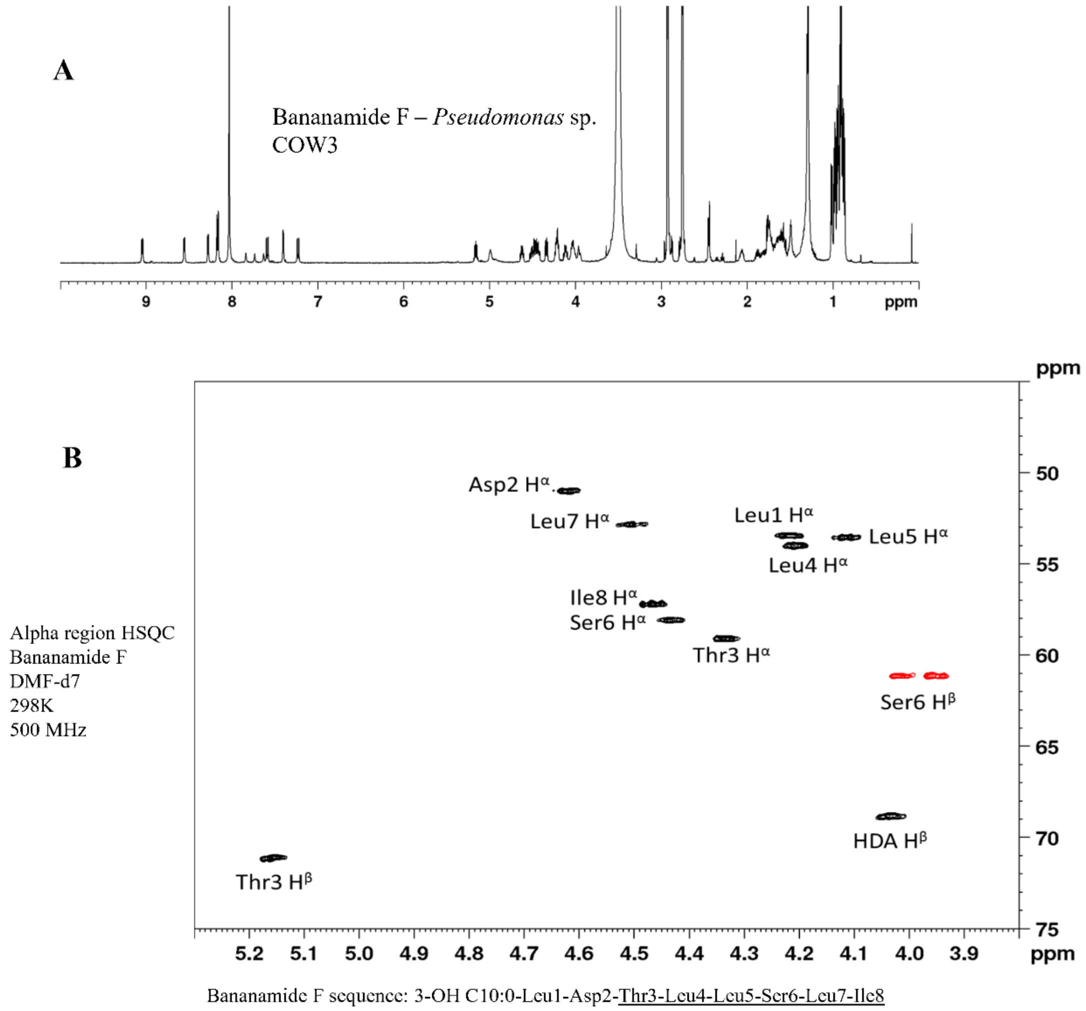

Figure S3. 1D ^{1}H NMR (**A**) and ^{1}H-^{13}C gHSQC spectrum (**B**) of bananamide F extracted from *Pseudomonas* sp. COW3 (DMF-d7, 298K, 500MHz). A. 1D 1H NMR spectrum of the first minor compound called bananamide F, eluting at 10.3 minutes. B. The alpha region of a ^{1}H-^{13}C gHSQC spectrum of bananamide F shows the presence of 8 amino acids. The high chemical shift of the Thr CH^{β} indicates that the C-terminal ester bond is formed with this residue.

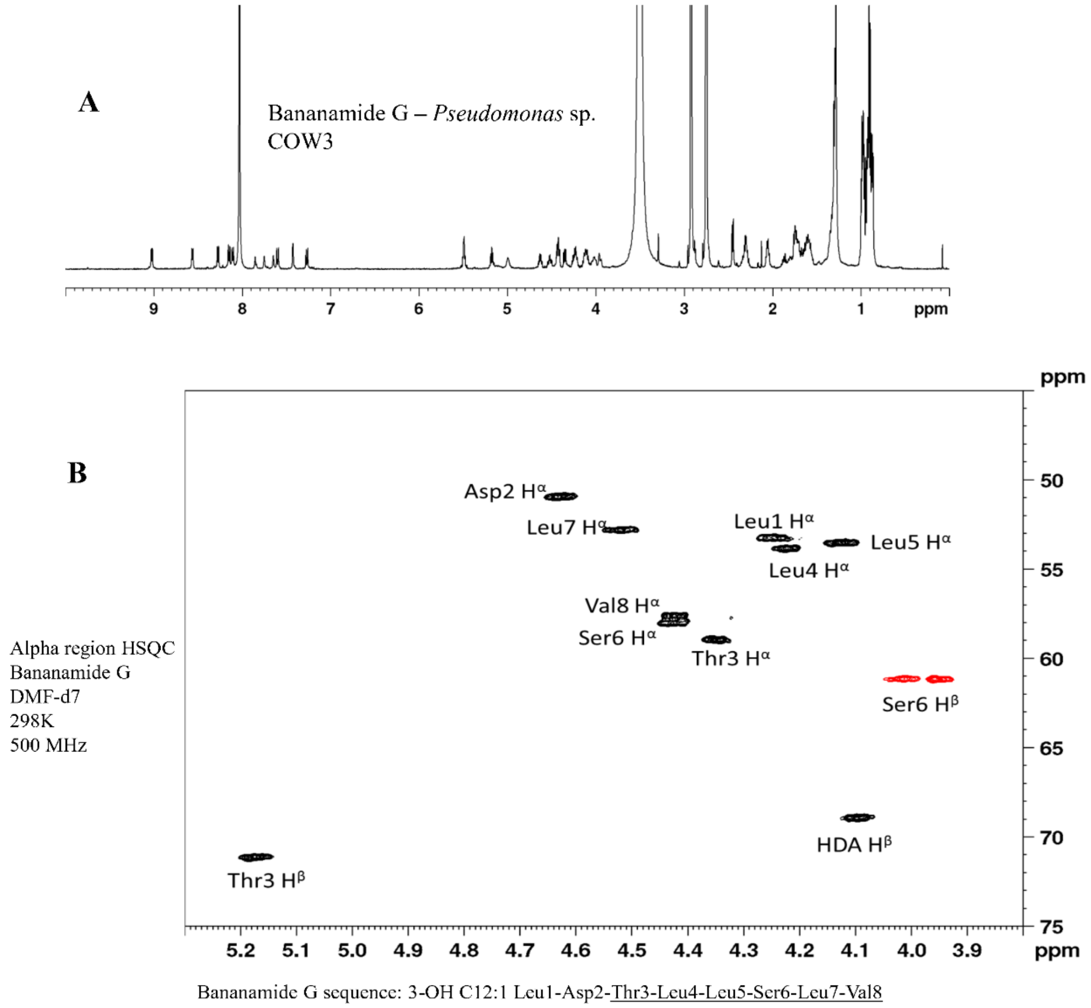

Figure S4. 1D ^{1}H NMR (**A**) and ^{1}H-^{13}C gHSQC spectrum (**B**) of bananamide G extracted from *Pseudomonas* sp. COW3 (DMF-d7, 298K, 500MHz). A. 1D 1H NMR spectrum of the second minor compound called bananamide G, eluting at 10.9 minutes. The presence of an unsaturation in the structure is immediately clear from the characteristic signal at 5.5 ppm. B. The alpha region of a ^{1}H-^{13}C gHSQC spectrum of bananamide G shows the presence of 8 amino acids.

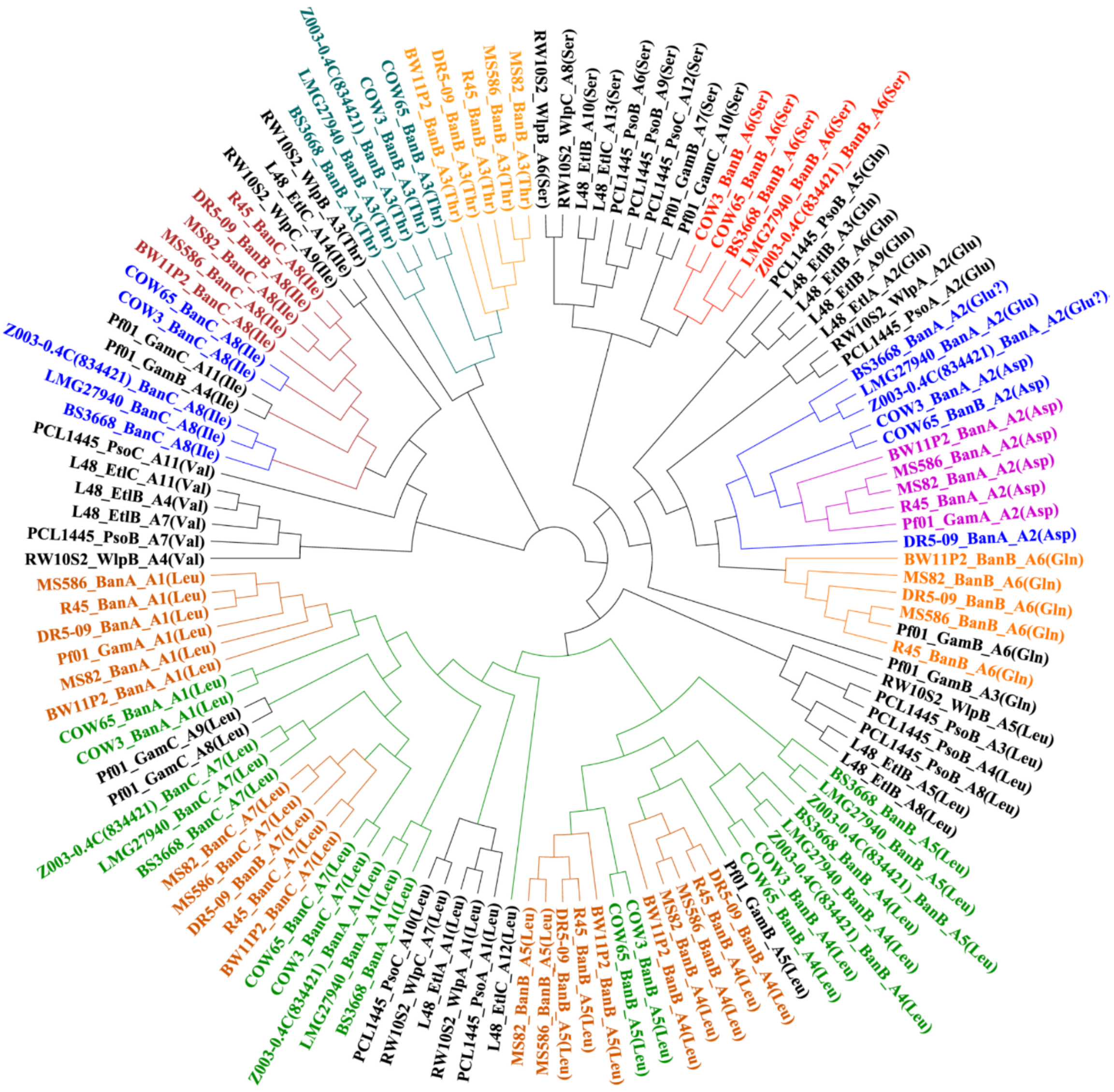

Figure S5. Phylogeny-based substrate specificity prediction of bananamide synthetases.
Cladogram of neighbor joining tree inferred from amino acid sequence alignment of adenylation (A) domains extracted from already characterized and putative *Pseudomonas* cyclic lipopeptides NRPSs in the bananamide group. Lipopeptide-specific codes are: Etl (entolysin, *P. entomophila* L48); Pso (putisolvin, *P. putida* PCL1445); Gam (gacamide, *P. fluorescens* Pf01); Ban (bananamide D-G, *Pseudomonas* sp. COW3); Ban (bananamide D-G, *Pseudomonas* sp. COW65); Ban (bananamide A-C, *Pseudomonas* sp. BW11P2); Ban (putative bananamide A-C, *Pseudomonas* sp. MS586) Ban (putative bananamide A-C, *Pseudomonas fluorescens* MS82); Ban (putative MDN-0066, *P. moraviensis* BS3668); Ban (MDN-0066, *P. granadensis* LMG 27940); Ban (putative bananamide A-C, *Pseudomonas* sp. R45); Ban (putative bananamide A-C, *Pseudomonas* sp. DR5-09); and Ban (putative MDN-0066, *Pseudomonas* sp. Z003-0.4C(8344-21). For each domain the substrate specificity is indicated in parentheses using the standard amino acid three-letter code. Clusters comprising bananamide domains are highlighted in different colors.

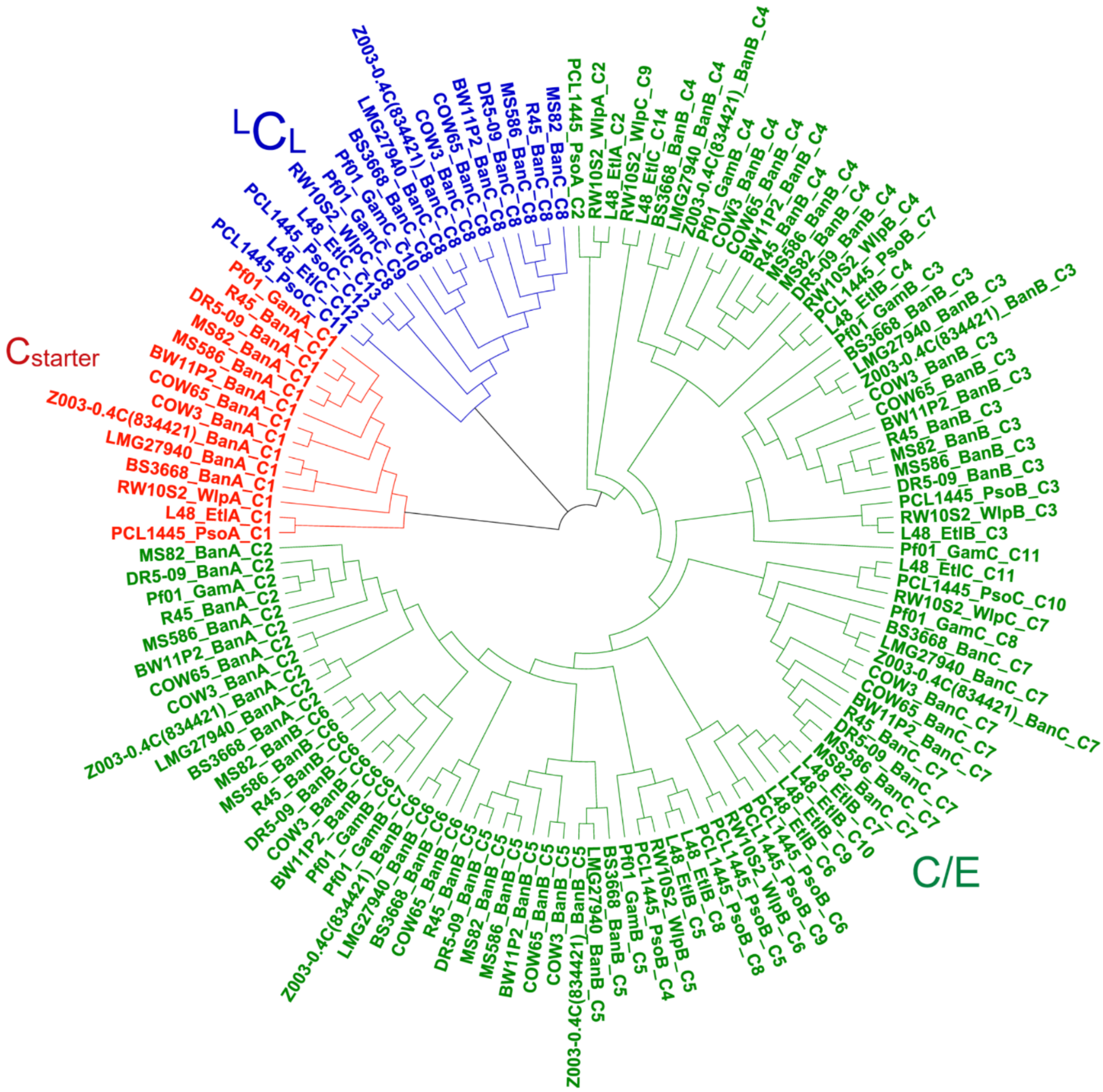

Figure S6. Phylogenetic analysis of condensation domains extracted from bananamide D-G, and from other putative bananamide-producing strains. Cladogram of neighbor joining tree inferred from alignment of condensation (C) domains extracted from already characterized and putative *Pseudomonas* cyclic lipopeptides NRPSs in the bananamide group. Lipopeptide-specific codes are: Etl (entolysin, *P. entomophila* L48); Pso (putisolvin, *P. putida* PCL1445); Gam (gacamide, *P. fluorescens* Pf01); Ban (bananamide D-G, *Pseudomonas* sp. COW3); Ban (bananamide D-G, *Pseudomonas* sp. COW65); Ban (bananamide A-C, *Pseudomonas* sp. BW11P2); Ban (putative bananamide A-C, *Pseudomonas* sp. MS586) Ban (putative bananamide A-C, *P. fluorescens* MS82); Ban (putative MDB-0066, *P. moraviensis* BS3668); Ban (MDN-0066, *P. granadensis* LMG 27940); Ban (putative bananamide A-C, *Pseudomonas* sp. R45); Ban (putative bananamide A-C, *Pseudomonas* sp. DR5-09); and Ban (putative MDN-0066, *Pseudomonas* sp. Z003-0.4C(8344-21).

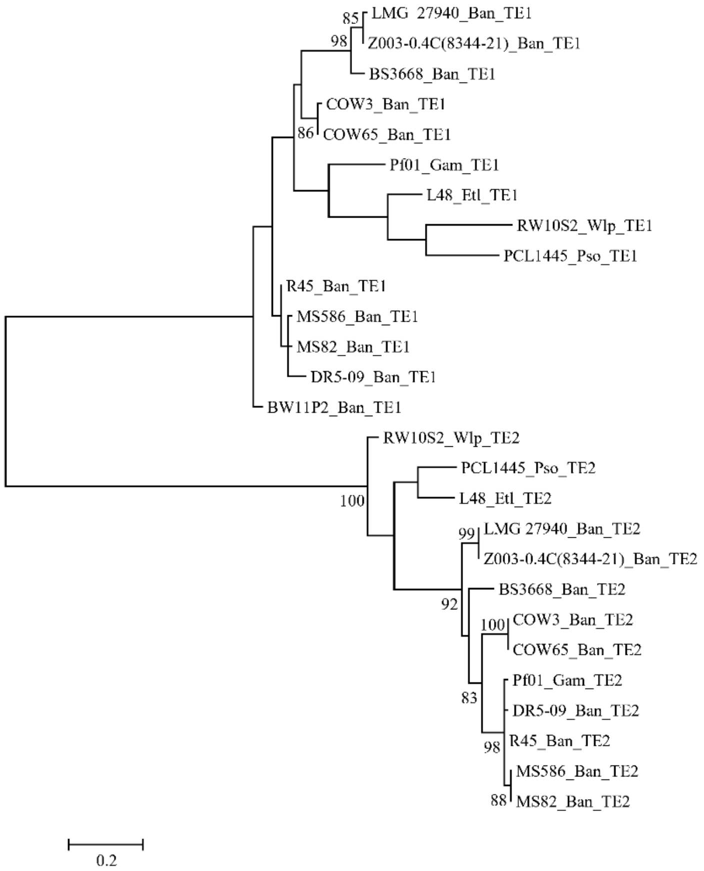

Figure S7. Thioesterase (TE) domain phylogenetic tree. Phylogenetic tree was constructed from thioesterase (TE) domains extracted from already characterized and putative *Pseudomonas* cyclic lipopeptides NRPSs in the bananamide group, with MEGA6 using the Maximum Likelihood Method with 1000 bootstrap replicates. Only bootstrap values above 70% are indicated. Lipopeptide-specific codes are: Etl (entolysin, *P. entomophila* L48); Pso (putisolvin, *P. putida* PCL1445); Gam (gacamide, *P. fluorescens* Pf01); Ban (bananamide D-G, *Pseudomonas* sp. COW3); Ban (bananamide D-G, *Pseudomonas* sp. COW65); Ban (bananamide A-C, *Pseudomonas* sp. BW11P2); Ban (putative bananamide A-C, *Pseudomonas* sp. MS586) Ban (putative bananamide A-C, *P. fluorescens* MS82); Ban (putative MDN-0066, *P. moraviensis* BS3668); Ban (MDN-0066, *P. granadensis* LMG 27940); Ban (putative bananamide A-C, *Pseudomonas* sp. R45); Ban (putative bananamide A-C, *Pseudomonas* sp. DR5-09); and Ban (putative MDN-0066, *Pseudomonas* sp. Z003-0.4C(8344-21).

Table S1. Novel bananamide synthetases and flanking region identified from *Pseudomonas* sp. COW3 and related strains in this study. The identity level of protein sequence was compared by BLASTp search.

	**Bananamide synthetases and flanking area**	***Pseudomonas* sp.**								
		**COW65**	**BS3668**	**LMG 27940**	**Z003-0.4C(8344-21)**	**BW11P2**	**DR 5-09**	**R45**	**MS586**	**MS82**
1	Organic hydroperoxide resistance protein	100%	-	-	-	-	93%<br>ANI54099.1	-	-	-
2	Copper metallochaperone, bacterial analog of Cox17 protein	100%	85%	-	88%<br>WP_093100925.1	88%	87%<br>WP_064595537.1	88%<br>WP_085748386.1	87%<br>AMQ85081.1	87%
3	Cytochrome oxidase biogenesis protein Sco1/SenC/PrrC, putative copper metallochaperone	99%	92%	-	-	92%	92%<br>WP_064595539.1	92%<br>WP_085748385.1	92%<br>AMQ85080.1	92%
nodT	RND efflux system, outer membrane lipoprotein	97%	78%	78%	78%<br>WP_093100911.1	86%<br>AOA33119.1	87%<br>WP_064595541.1	86%<br>WP_085748384.1	87%<br>AMQ85079.1	87%
luxR	LuxR family transcriptional regulator	99%	78%	78%	77%<br>WP_093100913.1	82%<br>AOA33120.1	84%<br>WP_064595544.1	84%<br>WP_085748383.1	83%<br>AMQ85078.1	83%
BanA	Non-ribosomal peptide synthetase	97%	73%	74%	73%<br>WP_093100915.1	82%<br>AOA33121.1	81%<br>WP_064595545.1	82%<br>WP_085748382.1	83%<br>AMQ85077.1	82%
BanB	Non-ribosomal peptide synthetase	95%	78%	78%	78%<br>WP_093100917.1	80%<br>AOA33122.1	80%<br>WP_064595547.1	80%<br>WP_085748381.1	80%<br>AMQ85076.2	80%
BanC	Non-ribosomal peptide synthetase	98%	80%	79%	79%<br>WP_093100919.1	84%<br>AOA33123.1	85%<br>WP_064595549.1	85%<br>WP_085748380.1	84%<br>AMQ85075.1	85%
macA	Macrolide efflux protein MacA	99%	91%	92%	92%<br>WP_093100921.1	93%<br>AOA33124.1	94%<br>WP_064595551.1	94%<br>WP_085748379.1	94%<br>AMQ85074.1	94%
macB	Macrolide efflux protein MacB	99%	93%	93%	93%<br>WP_093100923.1	93%<br>AOA33125.1	93%<br>WP_064595553.1	94%<br>WP_085748378.1	95%<br>AMQ85073.1	95%
luxR	LuxR family transcriptional regulator	99%	75%	74%	73%<br>WP_093106653.1	78%<br>AOA33126.1	81%<br>WP_064595555.1	80%<br>WP_085748377.1	81%<br>AMQ85072.1	79%
4	Glycosyl transferase, group 2 family protein	97%	-	-	-	-	-	-	-	

References: see Table A5
